# Modeling lexical abilities of heritage language and L2 speakers of Hebrew and English in Israel and the United States: a network approach

**DOI:** 10.3389/fpsyg.2024.1331801

**Published:** 2024-04-24

**Authors:** Clara Fridman, Adina Livni, Sagit Bar On, Natalia Meir

**Affiliations:** ^1^Department of English Literature and Linguistics, Bar Ilan University, Ramat Gan, Israel; ^2^Leslie and Susan Gonda Multidisciplinary Brain Research Center, Bar Ilan University, Ramat Gan, Israel

**Keywords:** heritage language, L2, English, Hebrew, network modeling, input factors, vocabulary, lexicon

## Abstract

**Introduction:**

This paper examines the productive vocabulary skills of five groups of English-Hebrew bilinguals in Israel and the United States. The juxtaposition of these five groups allows us to simultaneously compare performance across dominance profiles, acquisition contexts (L2 learned in school, HL maintained at home, immigration and immersion), and countries (Israel and the USA).

**Methods:**

A total of 185 participants took part in study: Hebrew-dominant heritage English speakers, Hebrew-dominant L2-English speakers, English-dominant heritage Hebrew speakers, and English-dominant L2-Hebrew speakers in the US and in Israel. They were all administered the MINT assessment in both languages, as well as background questionnaires. We then employ network modeling based on a secondary data analysis of background questionnaires to consider how each group’s lexical proficiency ties in to reported input factors.

**Results and discussion:**

The MINT results indicate clear language dominance in all the groups except Hebrew-dominant heritage English speakers, who show balanced proficiency in both their languages. The network models indicate key distinctions between the groups as a function of linguistic context, and we assess our findings in the context of recent work on quantifying the bilingual experience.

## 1 Introduction

Languages beyond the majority language spoken in a given society are acquired in a variety of ways. Under one scenario, a person acquires her first language (L1) in a monolingual setting, might learn a second language (L2) in school and become a late sequential bilingual, and this language acquisition might be supported to varying degrees by personal media consumption. In another scenario, a speaker might emigrate from one country to another, thus becoming immersed in a new L2 (which may or may not have been previously studied as an L2) as a late sequential bilingual or adult learner. While this latter scenario may be similar to the first example of L2 acquisition, the context—language acquisition in a classroom versus assimilation in everyday life—is crucially different. In yet another scenario, a speaker’s L1 might be her heritage language (HL), a language acquired naturalistically at an early age, distinct from the dominant societal language (SL) where the speaker grows up (Rothman, 2009). In this scenario, speakers start out either dominant in the HL or balanced in the HL and the SL, as either simultaneous or early sequential bilinguals, but shift dominance to the SL once they begin schooling (Benmamoun et al., 2013). While each of these manifestations of bilingualism—L2 acquisition in school, immigration contexts, and heritage languages—have been thoroughly studied in their own right, few studies have considered them in concert. These few studies have generally explored one language in various forms: as an HL, as an L2, and as a majority SL among monolingual speakers. Studies that consider these diverse bilingual scenarios across a language dyad are few and far between.

To bridge this gap, we combined production and language use data from five groups of bilingual English/Hebrew speakers, with some groups mirroring each other. These groups included Hebrew-dominant heritage-English speakers, Hebrew-dominant L2-English speakers, English-dominant heritage-Hebrew speakers, and English-dominant L2-Hebrew speakers in the US and in Israel (see the top section of Table 1 for a concise understanding of the groups and their language dynamic). We assessed the participants’ productive vocabularies in both Hebrew and English and conducted a network analysis of participants’ linguistic background and input factors and lexical proficiency in both languages. The goal of this paper is to compare the same languages in different contexts and with different statuses, and to explore how linguistic experience could play different roles accordingly. To this end, we purposefully compare a high-prestige language (English), which is a *lingua franca* (Phillipson, 2008), to a language with lower prestige but demonstrated cultural value (Hebrew), to consider how proficiency in each might interrelate with input and other factors. As the lexicon is known to be particularly susceptible to input factors (Gharibi and Boers, 2017), we expect to highlight multiple relations of interest between background variables and proficiency in Hebrew and English. The network analysis, described in greater detail in section 2.3, is a relatively novel methodology that allows us to explore potential connections between variables without explicitly testing for causation. Thus, the present study will shed light on the interconnectedness of lexical proficiency in English and Hebrew with other measures of language use, as a function of linguistic context. The present study combines data collected from three previous studies (Bar On and Meir, 2022; Livni and Meir, 2022; Fridman and Meir, 2023a), each using a unique background questionnaire. Therefore, in addition to juxtaposing proficiency scores and building network models, we will discuss the methodological implications and challenges of integrating cross-study data (see Nicklin and Plonsky, 2020; Bialystok et al., 2022 for previous examples of secondary data analysis).

**Table 1:** Participants’ background information.

	L2-ENG-IL	L2-HEB-US	L2-HEB-IL	HL-ENG-IL	HL-HEB-US
Country of residence	Israel	United States	Israel	Israel	United States
English status	L2	L1	L1	HL	SL
Hebrew status	L1	L2	L2	SL	HL
Number of participants	50	27	20	48	40
Age	21.6 (3.9) 18–30	22.1 (5.6) 18–36	26.3 (2.9) 23–30	21.9 (3.7) 18–30	26.3 (7.5) 18–44
Gender	26F, 24M	12F, 15M	9F, 11M	24F, 24M	15F, 25M
Age of onset of bilingualism	9.31 (2.09) 5–14	5.59 (2.31) 2.5–15	14.45 (4.37) 8–20	1.88 (1.91) 0–5	2.77 (3.03) 0–10

## 2 The bilingual lexicon

For several decades, bilinguals have consistently been shown to have a smaller vocabulary in each of their languages than monolingual speakers of either (Bialystok and Luk, 2012). If words are acquired as a function of frequency, then this phenomenon is a natural consequence of bilinguals splitting their time and exposure between two languages, while monolinguals’ time and exposure are concentrated on one, leading to lower frequency representations among bilinguals (Gollan et al., 2005).

A recent meta-analysis of 130 studies found evidence for the bilingual “lexical deficit” (Bylund et al., 2023, p. 898) only among sequential bilinguals who learned their L2 later in life, and only in the L2, and not for simultaneous bilinguals or for sequential bilinguals in their L1 (Bylund et al., 2023). Meanwhile, while HL speakers (who can be either simultaneous or early sequential bilinguals) score fairly consistently higher on vocabulary assessments in their dominant SL, usually their L2, than in their HL, their lexical proficiency has been shown to be highly variable (see, for example, Fridman and Meir, 2023a, findings that HL-Hebrew speakers in the US ranged from 15 to 82% accuracy on an HL vocabulary assessment). Generalizing beyond vocabulary size, studies have shown that knowledge of one language can affect the bilingual’s knowledge of another (Prior and van Hell, 2021), for example in cases of code-switching, co-activation, cross-linguistic influence, or other blending of features between languages at every linguistic level.

Grosjean (1989) thus argues that bilinguals are not, and cannot be considered as, “two monolinguals in one.” Counterpointing the documented limitations of bilingual vocabularies per language, some have posited that the conceptual vocabulary of bilinguals is quite robust. That is, even though bilinguals’ vocabulary knowledge is distributed across two languages, the number of concepts they can name in either of the languages matches that of monolinguals in their one language or even surpasses it (Oller et al., 2007; Core et al., 2013). Several studies of children have found bilingual conceptual vocabulary scores to be similar to or higher than those of monolinguals (Pearson et al., 1993; Junker and Stockman, 2002; Ehl et al., 2020). To our knowledge, no such studies have been conducted on bilingual adults, although we have no reason to believe this phenomenon would change significantly as bilinguals age.

Luk and Rothman (2022) note that, as with other linguistic domains, bilingual lexical abilities will vary by the type of bilingualism, the amount and type of language exposure, and many other factors. The sum of the findings discussed in this section points to the importance of considering language experience and background when assessing lexical proficiency, as a means to understand which factors play the most pivotal roles in promoting bilingual vocabulary.

### 2.1 The bilingual lexicon and effects of background and input factors

A variety of background and input factors have been proposed to predict lexical proficiency among bilinguals. Background factors, such as socio-economic status (SES) and biological age, and input factors, such as age of onset of bilingualism, number and types of interlocutors, and language use over time and across contexts, might have cumulative effects and/or interact with each other. We will discuss in this subsection how these factors affect language skills in bilinguals. Individuals’ socio-economic status (SES) also affects their language skills: children with lower SES have lower productive and receptive vocabulary skills. This has been demonstrated for monolingual and bilingual children, since SES might be a proxy for the richness of the language input available to the child (for an overview, see De Cat, 2021). In particular, higher parental education positively impacts both languages of bilingual children (Miękisz et al., 2017).

Finally, age is known to mediate L1 lexical development, as monolingual children know fewer words than monolingual adults. With respect to L2 and HL acquisition, the impact of age is not straightforward. A study of child and adult HL-Russian speakers found that adult HL speakers had a larger HL vocabulary than their child counterparts on verbs, while on nouns this was the case only for participants with SL-Hebrew, but not with SL-English (Fridman and Meir, 2023b). HL proficiency in children is often higher in early childhood and deteriorates as the HL speaker ages and becomes more exposed to the SL (Armon-Lotem et al., 2021). Thus, age is much more likely to interact with other factors, such as age of acquisition of the SL/L2 and cumulative input (for example, an older L2 speaker with an earlier age of acquisition of the L2 might have more cumulative input and therefore be more proficient or, conversely, might have had more time to forget vocabulary, if most learning took place earlier on and was not reinforced). Thus, these additional confounding input variables must be considered.

In addition to age, age of onset of bilingualism (AoB) generally refers to the age at which an individual becomes exposed to an additional language, whether this is the SL or the L2. It has been widely shown to play a key role in bilinguals’ language proficiency (Bylund et al., 2021). For L2 speakers, an earlier age of acquisition leads to higher proficiency in the L2, while for HL speakers, a later age of acquisition of the SL may lead to higher proficiency in the HL (Montrul, 2008; Paradis, 2023). Furthermore, HL speakers with a later AoB often have higher self-ratings of both HL proficiency and HL language use overall (Macbeth et al., 2022), although AoB does not always predict HL performance (Montrul and Sánchez-Walker, 2013). Thus, it becomes important to consider the interactions between AoB and language experience (Kim and Kim, 2022).

Language use in the family has been proposed to play an important role in HL maintenance and L2 acquisition. In particular, Bridges and Hoff (2014) found an effect of sibling language use on HL knowledge in children, such that children without older siblings at or above school-ages had more HL input than those with such siblings. This can be attributed to the fact that the older siblings begin switching into the more-widely-used SL and bring the SL home with them, using it to speak both with the younger HL speakers and with their parents, and in turn leading their parents to use it more, so that the younger HL speakers would have less HL (and more SL) parental input. Similarly, L2 input from older siblings was found to facilitate L2 development in children more so than did L2 input from the mother (Duncan and Paradis, 2020).

For studies of adults, while understanding language input from childhood may be informative, it may not correspond directly (or at all) to current real-world language use (Macbeth et al., 2022). For example, frequent exposure to the HL during childhood may have dwindled over time, and effects of language input from parents and siblings may fade once the HL speaker has moved out of the family home. Thus, it is important to consider current language use in addition to language use at different points in time, rather than using an aggregate average measure that lumps this language experience together without distinction.

Other input factors, such as HL use in the broader community, can directly contribute to HL speakers’ positive attitudes toward their HL, and in turn to higher HL proficiency (Jee, 2018). Motivations for maintaining and advancing HL proficiency include an intrinsic desire for easier communication with non-SL-speaking family members (Jee, 2018) but also extrinsic pressure from family (Comanaru and Noels, 2009). Some HL speakers see the HL as an innate part of their self-concept (*ibid*) while others report similar motivations to those of L2 learners: increased confidence, acquiring or sharpening a skill, or gaining a useful tool for career advancement. Studies have shown that L2 learners’ motivations to learn a language may be modulated by proficiency, with lower-proficiency learners citing general ideas such as participating in a multilingual workplace, and more advanced learners setting concrete goals for utilizing the language (Wen and Piao, 2020). Rodina et al. (2020) showed that the size of the HL-speaking community and access to formal instruction in the HL were significant predictors of HL performance in the domain of morphosyntax. Similarly, in a study of HL-Arabic speakers, Albirini (2014) found language use across time and contexts to be a significant predictor of HL proficiency, with the highest HL proficiency found among speakers with a higher number of interlocutors. In the same vein, it was shown that the lexical proficiency of HL-Hebrew, HL-Chinese, and HL-Spanish child speakers positively correlated with the number of HL interlocutors in the child’s environment (Gollan et al., 2015).

Several studies have found a positive effect of media consumption, focusing on television, on L2 vocabulary acquisition (Lin and Siyanova-Chanturia, 2015). Similarly, media engagement, as well as engagement in extracurricular and cultural activities, has been found to be positively correlated with bilingual vocabulary development (see Paradis, 2023 for an overview). Extending this, studies have shown a positive association between HL use with friends and HL proficiency (Paradis, 2023).

In some contexts, a language might be maintained as an HL or acquired as L2 for identity or religious purposes. Regarding identity, in a study of 40 college-aged HL-Korean speakers in the US, participants were overall found to have a high level of biculturalism—considering themselves a blend of Korean and American. Furthermore, participants with higher HL proficiency were more likely to rate themselves as bicultural, suggesting that greater proficiency in the HL leads to a greater ability to balance the societal and home cultures (Lee, 2002). Kagan (2012) likewise found that HL speakers tend to straddle the minority and majority cultures, often describing themselves as having hyphenated identities, such as Russian-American. Extending this, Albirini (2014) found a positive correlation between a strong sense of ethnic identity tied to the HL and HL-Arabic proficiency. Indeed, HL-Arabic speakers reported that they study Arabic in the USA for reasons of ethnic identity and because of their religious affiliation, be that Christian or Muslim (for an overview see Bale, 2010). The same can be applied to Hebrew, which is learned as part of the Jewish cultural and religious identity. For example, speakers of so-called Jewish English use thousands of words from Yiddish, Textual Hebrew/Aramaic, and Modern Israeli Hebrew (Benor, 2009, 2018) as part of their Jewish identity. The direction of the relationship between HL proficiency and identity is not always clear or unequivocal. While Lee (2002) interpreted HL-Korean proficiency as affecting self-identification, and Albirini (2014) suggested that identity inspired HL-Arabic speakers to formally study their HL, either of these cases could be interpreted in the reverse direction as well. Thus, until the observed correlations are studied in greater detail and in more contexts, we can only conclude that a connection has been identified between identity and HL proficiency, without commenting on causation.

### 2.2 Documenting diverse bilingual language experience: a network approach

The factors described in the previous sections have been considered across myriad studies and operationalized in numerous ways. Kašćelan et al. (2021) surveyed 48 different questionnaires aiming to profile language experience and background, finding a hefty range of factors of interest, methods for measuring those factors, and the scales employed therein. They note that even when the same labels are used across questionnaires, the variation within the measures begs the question of whether the same constructs are even being considered. For example, de Bruin (2019) points out that across numerous questionnaires, L2 age of acquisition might refer to the age at which a speaker immigrates to a new country, the age at which a speaker begins acquiring the language, the age at which a speaker reaches fluency in the language, the age at which a speaker becomes regularly exposed to the language on a daily basis, or the age at which the speaker begins receiving instruction in the language. Although there is consensus among the research community that age of acquisition is crucial in understanding an individual’s language background (de Cat et al., 2023), it is clearly apparent how the factor’s exact definition can have a significant effect on outcomes, and how defining it otherwise could lead to skewed results.

When considering current language use, it is not the operationalized definition that leaves room for doubt, but the formulation of the question (see Anderson et al., 2018 for an overview). This question might ask a bilingual participant to estimate the percentage of the use of one language per day, assuming that use of a second language makes up the difference to 100%. Alternatively, a participant might be asked to estimate an average daily percentage for each language, without assuming a maximum of two languages. Other questionnaires might consider the frequency of use of a given language, without defining a timeframe or setting, while still others might ask to note which languages were in use, specifying neither timeframe, nor frequency, but binary presence/absence. Naturally, the formulations of these questions leave significant space for variance and granularity and will play a significant role in the derivation of conclusions.

Furthermore, many questionnaires collecting self-rating information about language proficiency and use across contexts ask participants to provide an ordinal ranking. Setting aside the subjectivity of the estimations the participants provide, a more crucial issue is the variation in scales among these questions between questionnaires, with some prompting a ranking on a scale of 1–5, others of 1–7, others still 1–10, etc. It is not obvious whether a participant would provide an analogous ranking between the scales (Macbeth et al., 2022). For example, wanting to select an option close to, but not quite 100%, a participant might rank a 4 out of 5 on one scale, a 6 out of 7 on another, or even a 9 out of 10, leading to a variation of 5–10% for what was an estimate in the first place. Thus, comparing such self-assessments across varied questionnaires becomes problematic.

While there are undeniable practical challenges that come with assessing language data from questionnaires and all the more so from combining data collected from *different* questionnaires, the field is in agreement that bilinguals are far from a uniform population and can vary on myriad axes, each of which can be measured and indexed in a variety of ways (Kaushanskaya et al., 2020; Marian and Hayakawa, 2021; Kałamała et al., 2022a). Thus, in addition to challenges of interpreting questionnaire results, a task arises of how best to present and assess the interactions between this multitude of variables of interest. A recently proposed methodology for capturing the bilingual experience is network modeling (Freeborn et al., 2022; Kałamała et al., 2022b). Network modeling is most useful for assessing complex, dynamic, and multivariate systems which may not be explained as well through unidirectional statistical techniques (Zalbidea et al., 2023). Furthermore, this methodology is particularly useful as an exploratory model used to generate hypotheses based on estimated relationships and interdependencies (Epskamp et al., 2016), and is arguably more fitting than other methodologies such as structural equation modeling for the specific purpose of exploration (see Abacioglu et al., 2019 for a discussion). Network models have been used in related fields such as psychology for over a decade but have only recently begun to make waves in bilingualism research.

Network models consist of nodes, representing the variables entered into the model, and edges connecting these nodes, representing partial correlation coefficients between the variables (Bringmann et al., 2019). Edges can vary in density and color to represent the strength of the correlations and the direction of the relationships (positive or negative), respectively. It is important to note that, while network models can shed light on partial correlations between variables, they do not show causality, something that must be taken into account when interpreting findings. While network modeling can first and foremost show us the “bigger picture” understanding of which variables connect to each other and the ways in which they do so, we are also able to derive indices of centrality for each node, which can broadly highlight the influence of a given node within the network. The three most-commonly used measures of centrality are node strength, or the absolute number of connections a node has along with their robustness, betweenness, or how often a node can be found in the shortest path between two other nodes, and closeness, or how close the node is to other nodes (Zalbidea et al., 2023). However, the latter two measures have garnered significant scrutiny regarding their proper application and relevance in models beyond social networks (for example in the field of psychology), and it has been recommended that future work considers alternative measures or avoids them entirely (Bringmann et al., 2019). Bringmann et al. (2019) further draw attention to potential pitfalls of centrality measures, such as confounds that might arise among closely conceptually related factors (consider, for instance, the fundamental distinction between two nodes representing unique individuals in a social network as opposed to the two factors “language use between the ages of 6–12” and “language use between the ages of 13–18,” which have much clearer overlap). However, so long as the interpretations of centrality in a given model are made explicit, we believe that such a measure provides useful insights into particular relationships between particular nodes, and complement the network as a whole in showing us where we should point our efforts in future investigations.

### 2.3 The present study: English and Hebrew in Israel and the USA

The present work considers five groups from across the Hebrew-English dyad in Israel and the USA. Before outlining our research aims and hypotheses, it is important to set the stage for the context in which Hebrew and English are acquired.

In Israel, Hebrew is the SL, the only official language (for an overview of Israel’s linguistic makeup, see Meir et al., 2021). English enjoys a universal level of prestige in the country, although it is not one of Israel’s official languages (Gordon and Meir, 2023). In fact, English-dominant immigrants are less likely to attain the same levels of Hebrew as other immigrant groups, as their knowledge of English suffices to fulfill their major communication needs (Beenstock, 1996). English is one of seven required subjects for high school matriculation exams, and demonstrated proficiency is a prerequisite for university acceptance at all degree levels (Rose et al., 2023; Israeli Ministry of Education, 2024). English-speaking immigrants to Israel are typically well-educated and enjoy a relatively high socioeconomic status (Joffe, 2018). Most English-speaking families strive to speak mostly or exclusively English at home, in order to maintain or improve children’s English proficiency, as it is deemed important and advantageous for social and economic advancement (Kayam and Hirsch, 2012).

In the USA context, Israeli immigrants are considered a highly-assimilated and successful migrant group, although they are known to maintain close ties with their home country and are active in local expat communities (Fridman and Meir, 2023a). Notably, Israeli expats and their children are not the only speakers of Hebrew in the United States. Hebrew is also the ethnoreligious language used by diaspora Jews, both in religious and in cultural settings. For both groups, Hebrew is present through casual infusion or explicit instruction in Jewish day schools, Sunday schools, synagogues, and Jewish summer camps. Any venue or gathering with the purpose of connecting with Jewish heritage or culture will have formal or informal elements of Hebrew. While participation in cultural organizations is comparable, Israeli expats in the United States are as a group less religious than their non-Israeli Jewish counterparts (Rebhun and Lev-Ari, 2013). Thus, it is expected that American Jews will have greater exposure to and influence from Biblical and liturgical Hebrew. Notably, motivation for gaining (or maintaining) Hebrew proficiency likely differs between HL-Hebrew speakers and American L2-Hebrew learners, with the former group seeking to maintain a connection to family and homeland, and the latter aiming to find community and connection to their religion or to their ancestral heritage. This difference is likely not as pronounced among HL-English speakers and L2-English learners in Israel, for both of whom the motivation to improve English skills is likely driven by the international prestige and ubiquity of English. Today, English can be viewed as a *lingua franca* (Phillipson, 2008), as it is used worldwide for communication across a number of different domains, such as business, higher education, school settings and tourism (for an overview see Jenkins et al., 2011).

In the current study we investigate HL speakers of English in Israel and HL speakers of Hebrew in the USA, L2 speakers of English in Israel and L2 speakers of Hebrew in the USA, as well as L2 speakers of Hebrew in Israel. Our study had two central aims. We first set out to investigate how the five groups compare on lexical performance in both languages, as assessed by the MINT task (Gollan et al., 2012). We hypothesized that all groups would perform better in their SL than in their HL (for the HL groups) and in their L1 than in their L2 (for the L2 groups).

Next, we conducted an exploratory study to understand which background and input factors are most correlated between one another, and in particular with objectively measured lexical performance. The goal of this exploration was to understand how the inclusion or exclusion of particular factors would influence the network model, and to uncover interconnectedness between factors that are often viewed as unidirectional or unrelated predictors. Finally, with this work, we hope to contribute to the methodological discussion regarding the use of diverse or single background questionnaires.

## 3 Methods

The data and analysis script for this study can be retrieved from https://osf.io/p5ckj/?view_only=186fbcbb6f4e476bbea64e7b5f443627.

Data for the present study were compiled from the lexical proficiency and background data collected as part of three recently published and submitted studies (Bar On and Meir, 2022; Livni and Meir, 2022; Fridman and Meir, 2023a). A total of 185 adult participants were surveyed, making up five groups (see Table 1 for grouping definitions): 50 Israelis with L1-Hebrew who learned English in school as an L2 (L2-ENG-IL), 27 Americans with L1-English who learned Hebrew, in school or throughout extracurricular programs, as an L2 (L2-HEB-US), 20 Americans who learned Hebrew as an L2 and then moved to Israel (L2-HEB-IL), 48 Israeli heritage speakers of English, who were born in Israel or moved prior to age 4 and who came from English-speaking homes (HL-ENG-IL), and 40 American heritage speakers of Hebrew, who were born in the US or moved prior to age 4 and who came from Hebrew-speaking homes (HL-HEB-US). Group labels refer to the L2 or HL of each group and to their current place of residence (Israel: IL; the USA: US).

Participants’ lexical proficiency was assessed via the MINT assessment (Gollan et al., 2012) in both English and Hebrew, in decreasing order of proficiency. Additionally, participants completed a detailed background questionnaire about their language profile and practices. As the present study combines results from multiple others, the background questionnaires varied by group. Means, standard deviations, and ranges from measures available for all groups are compiled in Table 1.

### 3.1 Lexical proficiency: the MINT task

The MINT task (Gollan et al., 2012) contains 68 black-and-white line drawings that increase in naming difficulty, starting with simpler terms like “bear” and “clown” and ranging to more challenging words like “mortar” and “porthole.” When developing the MINT, Gollan et al. (2012) tested and calibrated it for use in research on bilingual speakers of Spanish, English, Hebrew, and Mandarin. In the current study, a participant response was counted as accurate even if the participant additionally named one or more inaccurate terms. Responses were thus binarily coded as “1” for a target response or “0” for a non-target response. Next, to assess conceptual vocabulary knowledge, we re-scored the same MINT results as follows. For each of the 68 items, a “1” was given if the participant accurately named the stimulus in at least one language; otherwise, the participant received a “0.”

### 3.2 Measuring background and language use factors: normalizing data across questionnaires

As the background and language use data assessed in this study were collected as part of three subprojects, three different questionnaires were used to assess background factors. These questionnaires were designed by their respective researchers, with no previous filiation. Furthermore, two of the groups, HL-ENG-IL and L2-ENG-IL, consisted of participants from two separate studies. Thus, only measures that were present in both studies’ questionnaires were taken into account for these groups. As a result, it can be said that four different background assessments were used in the present work. For convenience, we will be labeling these questionnaires A, B, C, and D. The L2-HEB-US group completed Questionnaire A, the L2-HEB-IL group completed Questionnaire B, the L2-ENG-IL and HL-ENG-IL groups completed Questionnaire C (the common factors of Questionnaires A and B), and the HL-HEB-US group completed Questionnaire D. For a full list of the variables collected from each questionnaire (see Supplementary material 1). One additional consideration for working across different pre-collected data was the use of different coding methodologies, especially for ranking language use. These distinct methodologies had to be normalized into one system, often leading to decreased precision in the final product (i.e., normalizing “how often do you use Hebrew at with your friends on a scale of never/rarely/sometimes/often/always” (as in Questionnaire A) with “which language(s) do you use with your friends on a scale of English/Both Hebrew and English/Hebrew” (as in Questionnaire D) and “which language(s) do you use with your friends on a scale of Hebrew/mostly Hebrew/both Hebrew and English/mostly English/English” (as in Questionnaire B) into the broadest common denominator).

Our network models run only on numerical values, so we included only ordinal and continuous variables, and we unified identical variables that used different scales. This was done in the following ways. Besides MINT scores, for two groups other language proficiency measures were available. Self-rated foreign-sounding accent when speaking Hebrew was coded on a scale of 1–7, with 1 being no detectable accent. For the L2-HEB-IL group, Hebrew level was calculated as a sum of self-ratings of reading, writing, comprehension, and speaking skills each on a scale of 1–7, with 1 being minimal skills and 7 being high proficiency, divided by the total possible score of 28. For the HL-HEB-US group, Hebrew and English levels were derived from self-ratings from 1 to 5, with 5 being the highest proficiency. Hebrew narrative performance was coded as the number of unique target tokens (in Hebrew) produced in a narrative (see Fridman and Meir, 2023a for an overview of the task). Parental and participant education level was measured in years.

Input measures were available in all the groups, yet different scales were used. In Questionnaire C, which combined data from the separate Questionnaires A and B, one study measured language use as “Languages mostly used with the given interlocutor,” coded as 1 for English, 2 for both English and Hebrew, and 3 for Hebrew, while the other asked participants to estimate the percent of time in which English was used with the given interlocutor, ranging from 1 (0%, never) to 5 (100%, always). These values were normalized to the scale of the first study, such that scores of 4 and 5 were coded as “English” or 1, a score of 3 was coded as “Both English and Hebrew” or 2, and scores of 1 and 2 were coded as “Hebrew” or 3. Thus, the resulting scale ordinally ranked Hebrew use (none or little—mixed—much or exclusive), using a coarser system than that of some of the original questionnaires. For clarity, and because participants command two languages such that the absence of one points to the presence of the other, we used the “English” label to indicate limited Hebrew use. This same scale was used to quantify language use at different age ranges in the HL-HEB-US group. For the HL-ENG-IL group, Hebrew age of acquisition was coded separately from Years (age) at Immigration, as many participants were not exposed to the societal language immediately upon arrival to Israel, up until the start of schooling.

In the HL-HEB-US group, we also collected information on participants’ identity and language maintenance. Maintenance methods were coded up to 10, with 1 point given to each of the listed methods for maintaining the HL. Visit frequency to Israel was coded from 1 to 5 (in ascending order: “I have not visited in the last decade,” “less than once every few years,” “once every few years,” “once a year,” “more than once a year”). Use of Hebrew in different speech contexts and with different interlocutors was coded from 1 to 5 (with 1 being “never” and 5 being “always”). Maintenance importance was coded from 1 to 5, and identity was coded as 1 for only American, 2 for Jewish, 3 for Jewish Israel-American, 4 for Israeli-American, and 5 for Israeli. For a full breakdown of the scales used for each variable, and for the full text of each questionnaire (see Supplementary materials 2, 5-8).

### 3.3 Statistical analysis

Data analysis for this study was done in two parts, each using R (R Core Team, 2021). First, MINT performance for both languages was compared across all groups. Using a linear mixed effects regression from the “lme4” R package (Bates et al., 2015), we assessed the effect of Age, Group, Language (English vs. Hebrew), and a Group × Language interaction on outcomes. Subsequently, we analyzed the conceptual vocabulary in the five groups. Conceptual vocabulary scores were also analyzed using a linear regression.

Next, using the R packages “bootnet” and “qgraph” (Epskamp et al., 2012, 2018) we built a network model for each group using the relevant background variables. Variables for which there was no variance (i.e., all participants spoke the same language with their mothers) or where not all participants in the group provided data (with the exception of variables such as “Language Use with Siblings” for which participants without siblings marked “N/A”) were excluded from the network analysis. We used the Gaussian Graphical Model (GGM) with Spearman partial correlations, which allowed us to account for a mix of ordinal and continuous variables (see Isvoranu and Epskamp, 2023). On the resulting model, we ran a centrality analysis to calculate the strength of each node.

As highlighted in section 2.3, network centrality analyses are controversial beyond social networks, and, if used, must be clearly defined in the context of a given study. Recall that node strength is measured as the sum of absolute values of relationships with consecutive nodes, taking into account both positive and negative correlation coefficients. Thus, the strongest node in the model is that which is connected to the greatest number of other nodes in the network. As the network does not inform on causality, we cannot assume that the strongest node is the most influential- or that it affects the most other factors- nor can we say that it is most affected by other variables. In the present paper, however, it can serve as an informative highlight of which factors to study further, as they seem to be connected *in some way* to many others. Here, we focus on two potential insights: first, an unexpectedly central node can underscore newfound relationships between linguistic background variables. Second, by considering a variety of models, we can see how the centrality of given nodes change with the exclusion and inclusion of different variables.

## 4 Results

### 4.1 Lexical performance

**Figure 1:**
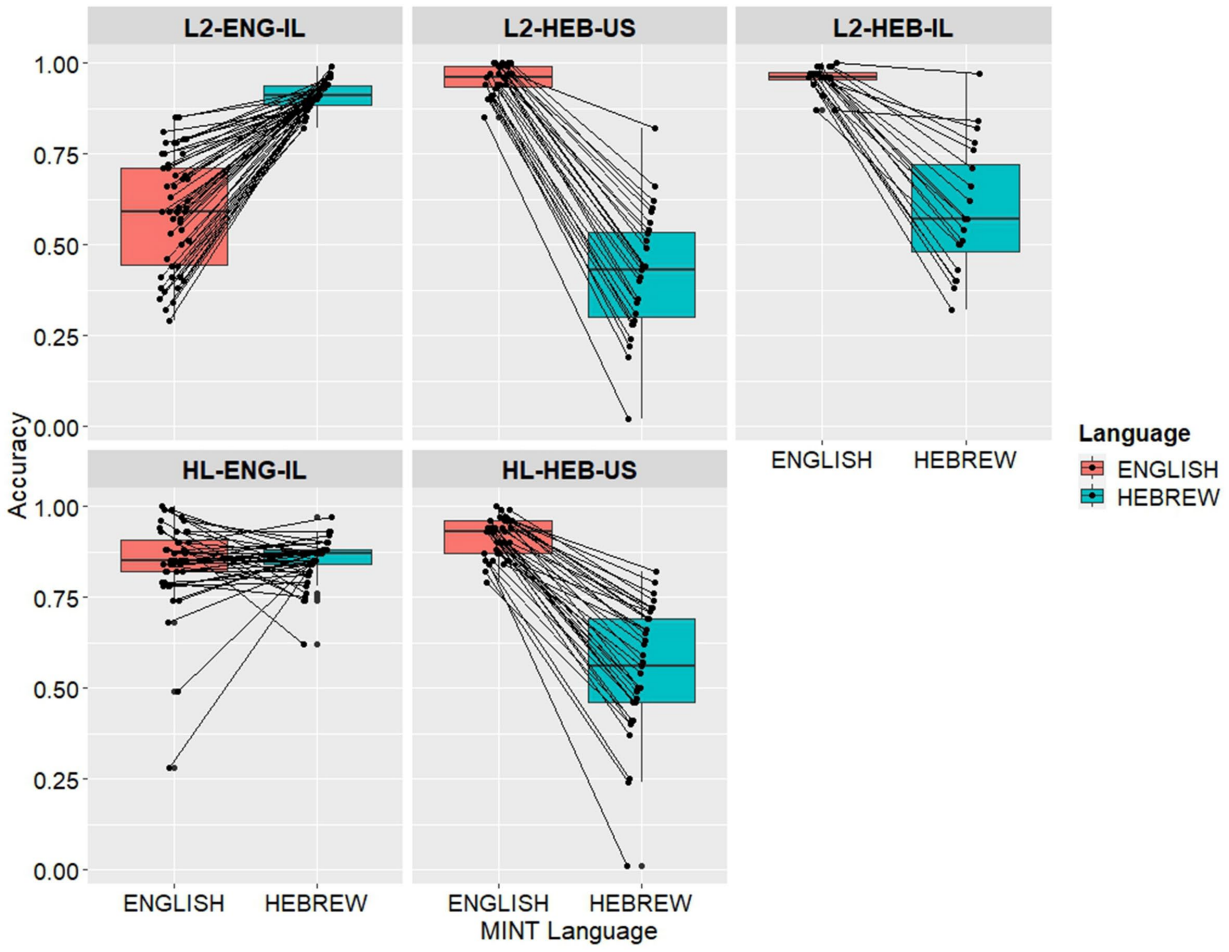
MINT scores of bilingual speakers across five groups. Each dot represents a MINT score and each line represents a participant, connecting the participant’s two scores.

Figure 1 shows the individual MINT scores for each group in each language. Each dot represents a participant’s score, and each line represents a participant, such that the balance/dominance between the two languages is clearly visible. The three lines on the boxplot represent (bottom to top) the first, second, and third quartiles, and the whiskers on either end of each box extend to the minimum and maximum value for each group. In all L2 groups and the HL-HEB-US group, one language is clearly dominant, with very little variance, while the other is clearly weaker, with a greater spread. By contrast, in the HL-ENG-IL group, we do not observe a clear dominance trend, with some participants performing more accurately in English and others in Hebrew, including several participants with very similar scores in both languages. This is especially notable in Table 2, which shows the means and ranges for each group and language, as well as the difference between the language means for each group. Here we can see that the HL-ENG-IL group had a 1% difference in dominance between the languages, while the L2-HEB-US group had a difference of 55%. The other three groups had a difference of 40–60%. Similarly, the range of each group in its societal language generally lies between 10 and 20%, while for the HL-ENG-IL group the range is at 35%.

**Table 2:** Means (SDs), ranges and differences of the English and Hebrew MINT scores.

	L2-ENG-IL	L2-HEB-US	L2-HEB-IL	HL-ENG-IL	HL-HEB-US
ENG MINT: M (SD)	0.59 (0.15)	0.95 (0.04)	0.96 (0.03)	0.84 (0.12)	0.92 (0.05)
ENG MINT: Range	0.29–0.85	0.85–1	0.87–1	0.28–1	0.79–1
HEB MINT: M (SD)	0.91 (0.04)	0.42 (0.17)	0.59 (0.17)	0.85 (0.06)	0.55 (0.16)
HEB MINT: Range	0.82–0.99	0.02–0.82	0.32–0.97	0.62–0.97	0.15–0.82
Mean ENG-HEB difference	0.32	0.53	0.37	0.01	0.37
ENG range	0.56	0.15	0.13	0.72	0.22
HEB range	0.17	0.80	0.65	0.35	0.67

We first ran a correlation analysis and found no significant correlation between Age and Age of Onset of Bilingualism (AoB), justifying our decision to separate the factors. Likewise, we considered combining these variables to consider “Length of Bilingualism” by subtracting AoB from Age; however, this variable was moderately to strongly correlated with each of the original two, so we kept the variables as originally collected. As an exploration to see how Length of Bilingualism would compare with its composite variables in explaining performance, we attempted to add it to a mixed effects regression model. However, with the addition of this variable, the model failed to converge, so we ultimately removed it and did not include it in further analyses. As there was little variance in AoB within each group, we did not include it in our model. As shown in Table 3, the results for the linear mixed effects regression, evaluating the contribution of Age, Group, Language, and a Group*Language interaction to the MINT scores, showed an effect of Age, such that older participants across all groups scored higher than younger ones, and effect of Group, no effect of Language and a significant Group*Language interaction. The Group* Language interaction was followed up by pairwise comparisons (see Table 4). The following pairwise significant group differences proved most notable. The HL-ENG-IL group matched the L2-ENG-IL group and the HL-HEB-US group matched both L1-English groups in each respective societal language. The HL-HEB-US group significantly outperformed the L2-HEB-US group on the Hebrew MINT (*p* < 0.0001), but not the L2-HEB-IL group, suggesting that the HL advantage over L2 learners diminishes with immersion. In this vein, the L2-HEB-IL group significantly outperformed the L2-HEB-US group on the Hebrew MINT (*p* < 0.0001). Subsequently, we also evaluated within-group performance, comparing English and Hebrew scores within each group (see Table 5). Four of the groups had a significantly dominant language out of the English-Hebrew pair. The HL-ENG-IL group was balanced between the HL and SL. Our analysis showed differences between the two languages in all groups except HL-ENG-IL.

**Table 3:** Linear mixed effects model for MINT performance across groups.

Predictors	Estimates	CI	Statistic	*p*
(Intercept)	0.76	0.70–0.82	23.93	**<0.001**
Age	0.00	0.00–0.01	3.21	**0.001**
Group [HL-HEB-US]	0.05	0.01–0.10	2.20	**0.029**
Group [L2-ENG-IL]	−0.25	−0.30 – −0.21	−11.16	**<0.001**
Group [L2-HEB-US]	0.11	0.06–0.16	3.99	**<0.001**
Group [L2-HEB-IL]	0.09	0.03–0.15	3.09	**0.002**
Language [Hebrew]	0.01	−0.04 – 0.05	0.33	0.738
Group [HL-HEB-US] * Language [Hebrew]	−0.37	−0.44 – −0.31	−11.50	**<0.001**
Group [L2-HEB-IL] * Language [Hebrew]	−0.37	−0.45 – −0.29	−9.24	**<0.001**
Group [L2-HEB-US] * Language [Hebrew]	−0.54	−0.61 – −0.47	−14.90	**<0.001**
Group [L2-ENG-IL] * Language [Hebrew]	0.31	0.25–0.37	10.24	**<0.001**

	Random effects
σ^2^	0.01
τ_00_ Participant	0.00
ICC	0.10
N_Participant_	185
Observations	370
Marginal R^2^/Conditional R^2^	0.712/0.742

The bold values represent significant values at *p* < 0.05.

**Table 4:** Pairwise between-group comparisons of English and Hebrew MINT scores.

Between-group comparison		*p*-value for English	*p*-value for Hebrew
HL-HEB-US vs. HL-ENG-IL		0.183	**<0.0001**
L2-ENG-IL vs. HL-ENG-IL		**<0.0001**	0.082
L2-HEB-IL vs. HL-ENG-IL		**0.018**	**<0.0001**
L2-HEB-US vs.	HL-ENG-IL	**<0.001**	**<0.0001**
L2-ENG-IL vs.	HL-HEB-US	**<0.0001**	**<0.0001**
L2-HEB-IL vs.	HL-HEB-US	0.698	0.688
L2-HEB-US vs.	HL-HEB-US	0.325	**<0.001**
L2-HEB-IL vs.	L2-ENG-IL	**<0.0001**	**<0.0001**
L2-HEB-US vs.	L2-ENG-IL	**<0.0001**	**<0.0001**
L2-HEB-IL vs.	L2-HEB-US	0.994	**<0.001**

**Table 5:** Within-group comparisons of English and Hebrew MINT scores.

Group	*p*-value
HL-ENG-IL	0.738
HL-HEB-US	**<0.0001**
L2-ENG-IL	**<0.0001**
L2-HEB-IL	**<0.0001**
L2-HEB-US	**<0.0001**

**Figure 2:**
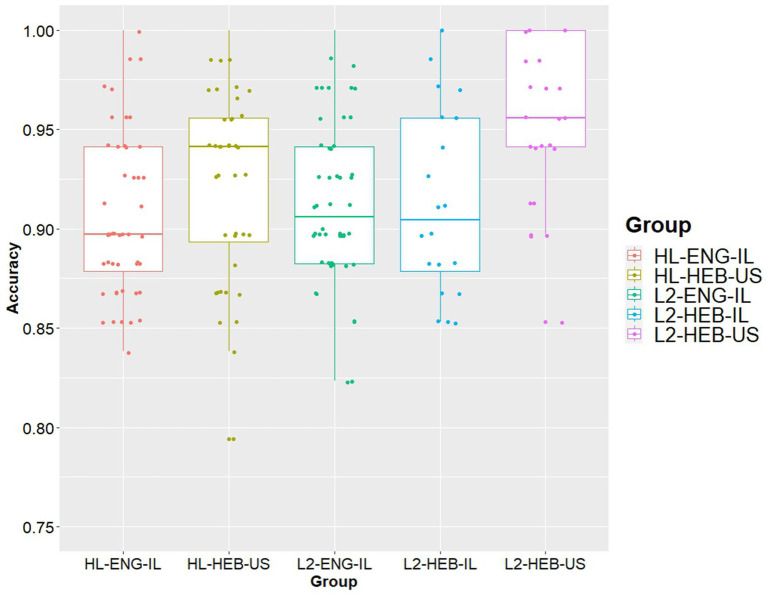
Conceptual vocabulary scores across five groups. Each dot represents a participant’s conceptual vocabulary score (the proportion of MINT items accurately named in at least one language).

To expand on group differences, we set out to evaluate each group’s conceptual vocabulary. Figure 2 shows the conceptual vocabulary sizes for each group, calculating the percentage of the total 68 items that each participant knew in at least one language. We can see that all groups have nearly at-ceiling performance, with no individuals from any group scoring below 75%. We then ran a linear mixed regression analysis on the data and found a significant effect of Group, with L2-HEB-US group scoring significantly higher (see Table 6). Following up on this effect with pairwise comparisons, we found that while the other four groups showed on par performance, the mean score for the L2-HEB-US group was significantly higher than that of any of the other groups. Notably, we did not find a significant effect of age, such that group differences persisted even with age entered as a predictor. Additionally, we compared conceptual vocabulary scores of each group per item (see Supplementary material 3) and found a wide between-group range in the later items of the MINT, which are meant to be more challenging.

**Table 6:** Mixed effects model for conceptual vocabulary.

Predictors	Estimates	CI	Statistic	*p*
(Intercept)	0.88	0.85–0.91	57.32	**<0.001**
Age	0.00	−0.00- 0.00	1.89	0.061
Group [HL-HEB-US]	0.01	−0.01-0.03	0.98	0.330
Group [L2-HEB-IL]	0.01	−0.02-0.02	0.01	0.994
Group [L2-HEB-US]	0.05	0.03–0.07	4.63	**<0.001**
Group [L2-ENG-IL]	0.01	−0.01-0.02	0.69	0.490
Observations	185			
R^2^/R^2^ Adjusted	0.136/0.112			

### 4.2 Network analysis

**Figure 3:**
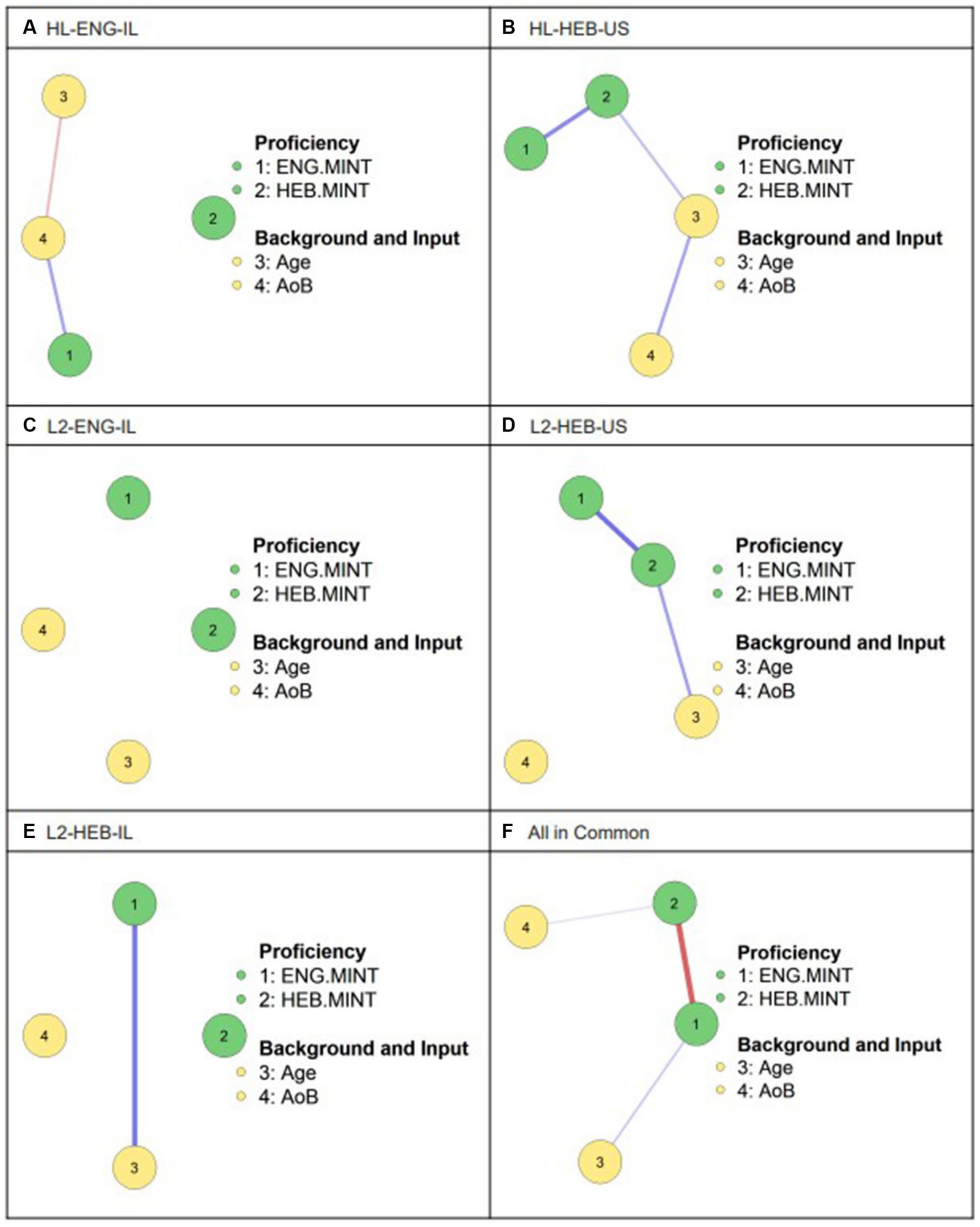
Network models for all groups using common factors. Each node represents a variable in the model. Each line represents a correlation between two factors, with red lines representing negative correlations and blue lines- positive ones. The thickness of the line represents the strength of the correlation. ENG.MINT = English MINT score, HEB.MINT = Hebrew MINT score, AoB = Age of Onset of Bilingualism.

**Figure 4:**
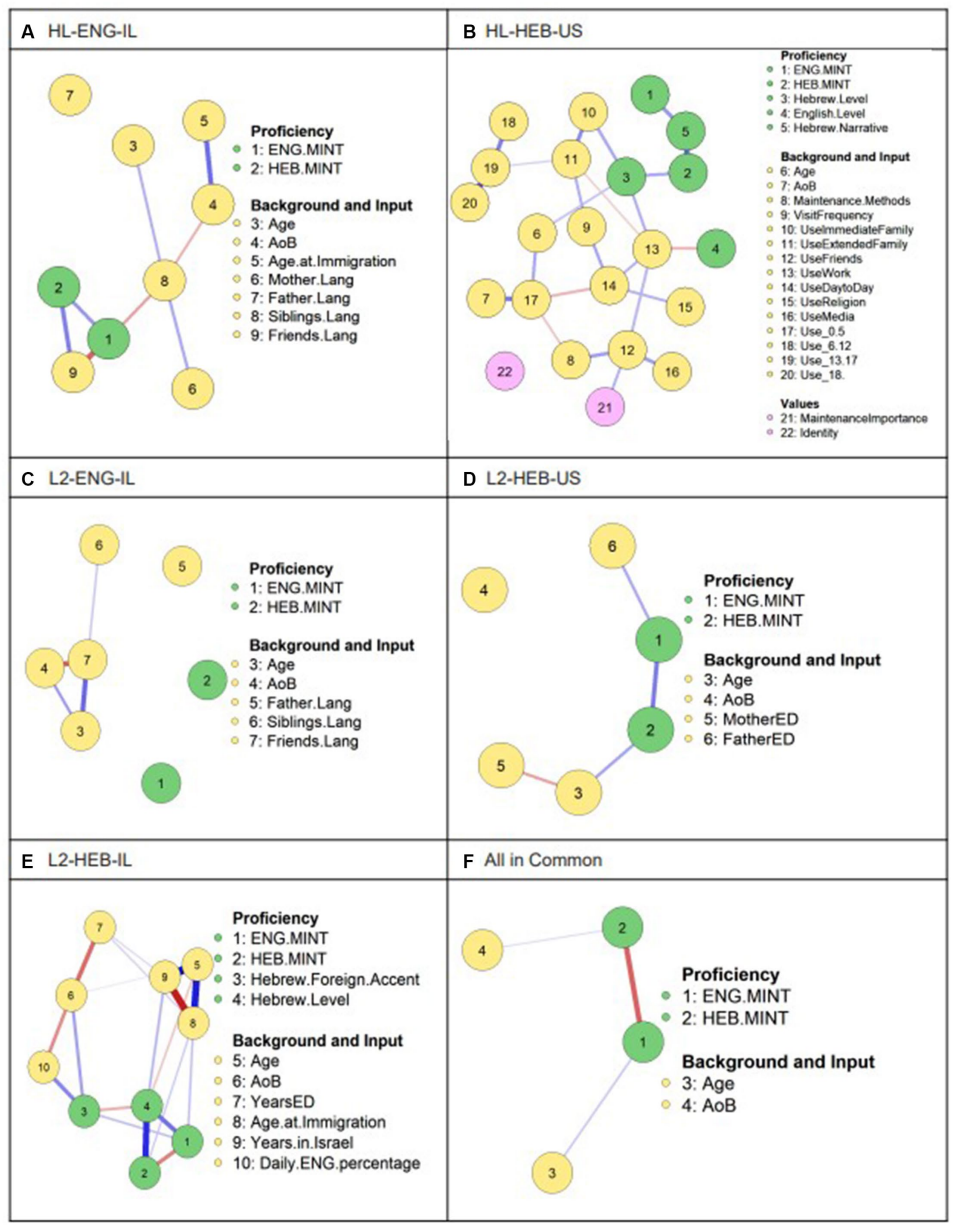
Network models for all groups using unique factors. Each node represents a variable in the model. Each line represents a correlation between two factors, with red lines representing negative correlations and blue lines- positive ones. The thickness of the line represents the strength of the correlation. ENG.MINT = English MINT score, HEB.MINT = Hebrew MINT score, AoB = Age of Onset of Bilingualism, Mother.Lang = Language Used with the Mother, Father.Lang = Language Used with the Father, Siblings.Lang = Language Used with the Siblings, Friends.Lang = Language Used with Friends, Hebrew.Level = Self-rated Hebrew Level, English.Level = Self-rated English Level, Hebrew.Narrative = the number of unique Hebrew tokens produced in a narrative task, Identity = cultural self-identification, MotherED = Mother’s Education Level in years, FatherED = Father’s Education Level in years, Daily.ENG.percentage = Percent of the day using English.

We conducted two network analyses for all groups. First, we built networks for each group using only those variables that were common to all questionnaires (Figure 3). Second, we built networks for each group using all the variables collected for that group specifically (Figure 4). In this way, we were able to compare the relationships between background and input factors as a function of the variables that were excluded or included. For both sets of network models, the nodes are color coded to distinguish between proficiency measures, background and input factors, and personal values (a category present only for the HL-HEB-US group that included the two variables: importance of maintaining the HL and cultural self-identification). The nodes are connected by blue or red lines, indicating positive or negative correlation coefficients, respectively, with line thickness representing the strength of the correlation coefficient. The absence of a line between a given pair of variables indicates the lack of a significant correlation between them.

Starting with Figure 3, which shows network models for each group using only the 4 variables available in all 5 groups, we found that in the two US-based groups, HL-HEB-US and L2-HEB-US, lexicon sizes in both languages were directly, positively related. For all Israel-based groups, no correlations were observed between vocabulary performance in each language. The model in Figure 3F collapsed all the participants into one group of “bilinguals”; here we saw an inverse relationship between English and Hebrew performance. This, of course, is perfectly expected considering that this model would group together many unbalanced speakers for whom one language would be much stronger than the other, culminating in this inverse correlation. However, we mention it to highlight that, without accounting for linguistic context and language dynamics (i.e., heritage bilingualism vs. second language acquisition vs. immersion), we lose the nuances demonstrated in the separate group models and could come to very different conclusions about the relationships between lexicons.

In the HL-HEB-US and L2-HEB-US groups, a positive correlation was found between age and performance on the MINT in the weaker language. Thus, older participants scored higher on the HL/L2. Conversely, in the immersion context, older participants from the L2-HEB-IL group scored higher on the MINT in their dominant L1. In the L2-ENG-IL group, age was not correlated with any of the other variables in the model. In the collapsed bilingual group in Figure 3F, age is shown to positively correlate with English performance, not accounting for the status of English as an HL or an L1. Note that there were only 20 participants in the group that showed a connection between English production and age, with 67 in those that showed a connection between Hebrew production and age, and 98 in groups that did not find connections among these variables. Nonetheless, the overall bilingual model presented a relationship between age and English production, further highlighting the extent to which the whole cannot be considered to be the sum of its parts. Finally and intuitively, in the HL-ENG-IL group, later age of onset of bilingualism (AoB) was correlated with better performance on the HL. Meanwhile, in Figure 3F, AoB is positively correlated with Hebrew performance, a relationship not found in any of the individual models from Figure 3.

Ultimately, few meaningful generalizations can be found from this comparison, primarily due to the small number of factors that were common across all groups. Even if we were to find a consistent pattern of intervariable relationships in all of the groups, too many pieces of the language experience puzzle are missing to be able to draw insightful conclusions. We thus sum up the preliminary findings of this set of networks as follows: in the US, older participants perform better on the HL/L2. In Israel, HL-English speakers who acquired Hebrew later, as well as older L2-Hebrew-speaking immigrants, have higher English scores. Finally, neither age nor AoB affect vocabulary scores in either language of Israeli L2-English speakers. Having observed these correlations, we now consider a new set of network models in Figure 4, which include a larger set of variables for each group.

When considering all of the available factors for each network, we found a direct relationship in the lexicon sizes in the dominant and non-dominant languages in 3 out of 5 groups (L2-HEB-US, L2-HEB-IL, and HL-ENG-IL). In the L2-HEB-IL group, the relationship was inverse, with higher scores on the English MINT correlated to lower scores on the Hebrew MINT, while in the remaining groups, scores on the two MINTs were positively correlated. In the HL-HEB-US group, the MINT nodes are both modified by the Hebrew narrative node, indicating high correlation coefficients between lexical proficiency in both languages and the ability to produce a high number of Hebrew tokens in a free speech elicitation task. In both the HL-HEB-US group and the L2-HEB-IL group, Hebrew MINT scores were highly positively correlated with overall Hebrew self-ratings. Interestingly, in the latter group, Hebrew self-ratings were also positively correlated with performance on L1 English production. Additionally, L2-HEB-IL participants with higher English MINT scores rated themselves as having more of a foreign accent in Hebrew (alternatively: participants who reported having more a foreign accent in Hebrew also scored higher on the English MINT). In the L2-ENG-IL group, as had been the case in the reduced model from Figure 3C, the two lexicon sizes were not related.

Looking at background and input factors, a positive correlation was found between age and L2 production in the L2-HEB-US group, echoing findings from the smaller network in Figure 3D. Age was also positively correlated with Hebrew self-ratings in the HL-HEB-US group, but not to MINT performance, contrasting findings from Figure 3B. By contrast, age was inversely correlated with Hebrew self-ratings in the L2-HEB-IL group, although, as in Figure 3E, age correlated positively with performance on the English MINT. As for SES, which was measured as the level of education of participants and/or their parents, in the L2-HEB-US group, the father’s years of education positively correlated with L1 lexicon size.

We found no correlation in any group between AoB and MINT performance in either language. This contrasts the network in Figure 3A, where this factor positively correlated with HL performance. In the L2-HEB-IL group, participants with later ages of Hebrew acquisition rated themselves as having a stronger foreign-sounding accent in Hebrew. In this group, age at immigration (measured separately from age of acquisition in this group as discussed in Section 2.3) was positively correlated with production on both MINTs, such that participants who moved to Israel at a later age scored higher. Furthermore, those who had been in Israel longer gave themselves higher Hebrew self-ratings, and those who used more English daily noted that they had a stronger foreign-sounding accent in Hebrew. Language spoken with the father was not correlated with any other nodes in the models for both the L2-ENG-IL and the HL-ENG-IL groups. Likewise, language spoken with the mother did not correlate with proficiency in the latter group. In the HL-HEB-US group, language use with the immediate family and at work correlated with self-rated Hebrew level, such that participants who used more Hebrew with their immediate family and/or at work gave themselves higher ratings. Language use with siblings and language use with friends correlated with English production in the HL-ENG-IL group, such that participants who used more English with their siblings and/or their friends scored higher on the English MINT. By contrast, language use with friends did not correlate with any proficiency measures in the HL-HEB-US group. While the input factors measuring visit frequency to Israel, the number of different methods used to maintain Hebrew, as well as Hebrew use with extended family and with friends, for religious services and media consumption, and during day-to-day interactions were all closely interconnected, none were correlated to lexical proficiency. Self-identification (as fully Israeli, American, a hyphenated variety, or otherwise) was unrelated to any of the other nodes in the model.

We compare these new models (Figures 4A–E) with Figure 4F, which, as it had in Figure 3F, combines all groups into one and considers only the variables that all groups have in common. Here, again, we find an inverse relationship between Hebrew and English MINT scores, despite only one group- the immersed L2-HEB-IL group with only 20 participants- exhibiting such a correlation, and all other groups showing either a direct positive relationship between MINT scores, a modulated one, or none at all. We further see a positive correlation between age and English MINT performance, despite only one group- the same aforementioned L2-HEB-IL group- having shown such a correlation. AoB was also found to positively correlate with Hebrew MINT scores, although no such correlation was observed in any of the other groups. While the model in Figure 4F did not yield any surprising results, we mention it here to highlight the importance of considering language background when looking at bilingual groups, as demonstrated by the fact that the combined model hardly resembles any of the models from individual groups.

Finally, we considered the centrality measure of strength (see Supplementary material 4) to understand which node in each model from Figure 4 was the most connected to other variables, whether positively or inversely. Note that node strength only takes into account direct relationships, rather than modulated ones. Each of the five groups had a different strongest node. In the HL-ENG-IL group, the strongest node was performance on the HL-English MINT, which was connected to Hebrew MINT performance as well as language use with siblings and friends. In the HL-HEB-US group, the strongest node was language use prior to age 5, which was connected to age, AoB, maintenance methods and day-to-day use. In the L2-ENG-IL group, the strongest node was language use with friends, which was connected to language use with siblings, biological age, and AoB. In the L2-HEB-US group, the strongest node was Hebrew MINT performance, which correlated with English MINT performance and age. Finally, in the L2-HEB-IL group, the strongest node was age at immigration, which was connected to biological age and number of years in Israel, years of education, and performance on both MINTs. The least central nodes also differed across the five groups, and included language use with the father, cultural self-identification, English MINT score, AoB, and years of education.

## 5 Discussion

The present study set out to explore lexical production of five groups of bilinguals across the Hebrew-English dyad, comparing heritage bilingualism and second language acquisition in different contexts, as well as the interrelatedness between lexical proficiency and background and input factors in their bilingual experience.

### 5.1 Lexical proficiency in Hebrew and English: dominant vs. non-dominant languages (L1 vs. L2; HL vs. SL)

We first considered lexical abilities in the two languages of each bilingual group, as measured via MINT assessments, and found that all but one had a clear dominant language. All the L2-speaking groups were dominant in their L1, and the HL-HEB-US group was dominant in their SL. The HL-ENG-IL group, however, was balanced in their HL and SL proficiencies, an unusual phenomenon for HL speakers. Consider, for instance, that one of the seminal works characterizing HL speakers adds as a definition that “Heritage speakers have as their dominant language the language of the host country” (Benmamoun et al., 2013, p. 132). The notion of bilinguals generally, not just HL speakers, having a dominant language rather than being fully balanced, has also been widely accepted as a given (Montrul, 2016).

The balance between the HL and SL in the HL-ENG-IL group could be due to a blend of possible contributing factors. First is the unique status of HL-English, both in general and specifically in Israel (Phillipson, 2008; Gordon and Meir, 2023; Rose et al., 2023). As discussed in section 2.4, knowledge of English is considered a notable asset in Israeli society, denoting a level of prestige and providing ample opportunities for academic and economic advancement. Because of this (or, perhaps, leading to this outcome), English-dominant immigrant parents make significant efforts to maintain and improve their children’s HL-English skills. HL-English speakers have been shown to “control extensive vocabulary” and be “familiar with an everyday lexicon that takes L2 learners’ years to acquire” (Polinsky, 2018). Furthermore, English is taught in schools, such that HL-English speakers would receive the same level of instruction as L2-English speakers and the same level of societal prevalence of English, with the only differences being age of acquisition and input and exposure in the home. It is unclear from the present study to what extent the unique performance of the HL-ENG-IL group is caused by greater language input from an earlier age, additional HL support in school instruction, and/or the status of the particular language in society. Therefore, it would be worthwhile to conduct a similar experiment in a different linguistic context and control for these variables: is this finding consistent for HL-English speakers around the world? Could it be replicated for HL speakers of different languages who also receive supporting instruction?

Additionally, we found that at the group level, HL speakers outperformed L2 speakers of each respective language in the same country, supporting findings across linguistic domains that found an advantage for HL speakers over L2 learners in oral production (Montrul et al., 2008; Saadah, 2011; Albirini and Benmamoun, 2014; Rakhilina et al., 2016). Likewise, this finding seems to support the results of the meta-analysis by Bylund et al. (2023), who found an advantage for simultaneous bilinguals (in this case HL speakers, although several of them were, in fact, sequential, although not by a significant number of years) over sequential bilinguals in the L2/HL, but not in the L1/SL. Notably, however, the HL-HEB-US group matched the L2-HEB-IL group on the Hebrew MINT, suggesting that the HL advantage over the L2 learner disappears in an immersive environment. We predict that this is related to the increased amount of input in the target language for the latter group, as compared to the former.

Finally, we explored the conceptual vocabulary of the five groups, to see whether each set of bilinguals had a conceptual representation of the tested lexical items in at least one language. Here, we found that the L2-HEB-US group scored significantly higher than each of the other four groups, which were otherwise matched among themselves. We suggest two possible explanations for this finding. First, the L2-HEB-US group is significantly older than the other two groups, with a mean age of 30 compared to the others’ 21 and 26, although the age range is the same among the groups. Similarly, the mixed effects model in Table 6 shows no significant effect age overall, such that group differences persist even when age is taken into account. Notably, this mean age aligns with that of the original group of bilinguals tested by Gollan et al. (2012), who were 19 on average at the time, and would today be around age 30. Thus, it could potentially be argued that the MINT is skewed toward this particular age group, with certain items being more familiar to older participants.

Another plausible explanation is that perhaps the MINT is not, in fact, “relatively culture neutral” (Gollan et al., 2012, p. 598), but rather particularly geared toward the American context, as has recently been suggested in a study of monolingual Chinese speakers that used an abbreviated version of the MINT (Li et al., 2022). Take, for instance, Item 68 - axle- which 64% of the L2-HEB-US group could identify in at least one language, but which only 8% of both the HLE-SLH-US and the L2-ENG-IL groups could. We pay particular attention to comparisons between the conceptual vocabulary of the L2-HEB-US group and its parallel L2-ENG-IL group. For the last 8 items of the MINT, the former group outscores the latter by anywhere from 5 to 50%, suggesting that these target words are not equally familiar in Hebrew as they are in English. These findings lead us to question the validity of the MINT assessment for a Hebrew-speaking context. The cultural neutrality explanation might hold for the groups raised in Israel, but does not sufficiently predict why the L2-HEB-US group would also outperform the US-raised groups, HL-HEB-US and L2-HEB-IL. For the former group, it can be argued that their upbringing in a mixed-culture or immigrant home, and thus the SL lexicon to which they are exposed, may qualitatively differ from their non-Israeli peers. However, this would not be the case for the L2-HEB-IL group. Nonetheless, we see that there are some items, such as “hinge” or “anvil,” that were identified by over 20% more L2-HEB-US participants than L2-HEB-IL participants. Therefore, we propose that the L2-HEB-US advantage stems from a cumulative effect of both older age and a cultural skew from the MINT. We thus encourage future iterations of the MINT assessment to test a wider generational and cultural span.

### 5.2 Network modeling: interconnected relationships between lexical proficiency and background and input factors

We next built network models of lexical proficiency and background and input factors for each group, followed by a summative model of bilinguals for all participants together. Our goal was to explore how these measures interact, and how this interaction changes both between groups and in combination with different factors, as there was no singular questionnaire used for all participants.

We first considered models for each group using only the four measures common to them all: English MINT score, Hebrew MINT score, Age, and Age of Onset of Bilingualism (AoB). We found that in both US-based groups, performance on the two MINTs was positively correlated and older participants scored higher on the weaker Hebrew MINT, while in the immersed L2-HEB-IL group, older participants scored higher on the English MINT. In the latter group, this finding could be tied to the fact that older immigrants may be less integrated into the dominant-language-speaking society, leading them to join enclaves of similar immigrant speakers, a trend that is particularly salient in Israeli so-called “anglo-communities” (Beenstock, 1996). The finding in the US-based groups, however, is trickier to interpret, as we cannot extrapolate from participant age any of the often-related factors that we explicitly considered in these groups, such as AoB, motivation to maintain the weaker language, or input in youth. Furthermore, while these factors might be expected to affect HL speakers, they are less obvious for the L2 group, for whom the weaker language is learned later and not explicitly supported in the home. In the HL-ENG-IL group HL-English MINT performance was tied to a later AoB. This is notable less for the finding itself, as it is quite reasonable to expect that HL speakers with longer uninterrupted exposure to the HL will have higher proficiency, but rather for the *absence* of this finding in other groups- especially the HL-HEB-US group. Together, these findings therefore further emphasize the role of particular language context, as not all HL, or in fact bilingual, networks paint the same picture, even when the same variables are considered. Collapsing all of the participants into a single bilingual group, age was found to be positively correlated with English MINT performance, although such a relationship had been observed only in one of the five groups, and the smallest group at that. AoB was correlated with Hebrew performance, although this correlation had not been found in any of the individual groups, raising further suspicions about the utility of such a collapsed view of bilinguals without consideration for context.

From this limited set of network models, we can already see a distinction in variable relationships based on linguistic context. When we added additional factors to each individual group’s network (Figure 4), some of the above relationships morphed while others were preserved. This shift can be likened to one that can be observed in other forms of analysis, such as regression models, where the addition of new predictors can affect the significance of others. Furthermore, it points to the importance of carefully considering which variables will or will not be included in the model, as interpretations and insights will largely depend on the selected parameters. As in the common network models, lexicon sizes between the two languages were directly connected in the L2-HEB-US group. However, with the addition of new variables, a correlation emerged between lexicon sizes in the HL-ENG-IL and L2-HEB-IL groups as well, with the former correlation being positive and the latter inverse, suggesting an effect that emerges in an immersive environment. This finding ties in to known effects of immersion on L1 lexical access, wherein as frequency of L1 use decreases (due to L2 immersion), lexical access is hindered (Baus et al., 2013; Steinhauer and Kasparian, 2020). Meanwhile, the direct correlation between lexicon sizes in the HL-HEB-US group that had been observed in the smaller models disappeared in favor of modulation by Hebrew narrative performance. Across all groups that had them, MINT scores were also strongly associated with other proficiency indices including self-ratings in the weaker language (echoing results from Gollan et al., 2012; Macbeth et al., 2022, who found strong correlations between assessed and self-rated HL proficiency), foreign accent ratings, and narrative skills.

Next, we found that age of immigration was positively correlated with MINT scores in both languages in the L2-HEB-IL group. This appears to be a counterintuitive finding. It is logical that those who immigrated at later stages of adulthood would be highly proficient in their L1. However, it is then unclear why those who immigrated at a later age would also have higher L2 scores in the immersion setting, and this is a potential point for future investigation.

The questionnaire for the HL-HEB-US group investigated, among other input factors, the frequency of Hebrew use with the immediate family, and the model showed that this factor was positively correlated with self-rated Hebrew level, but not with any of the MINT scores. Meanwhile the questionnaire for the L2-ENG-IL and HL-ENG-IL groups split this category into language use with the father, mother, and siblings. Language use with the father was found to be completely uncorrelated with any of the other nodes in either model, while language use with the mother was connected to other input measures, but not to proficiency. Meanwhile, in the HL-ENG-IL group, more English use with the siblings correlated with higher scores on the English MINT. This leads to a methodological question: what is the benefit of splitting the measure of language use with the immediate family into three separate components? In the present case, separating the measures allowed us to pinpoint which interlocutors within the immediate family had the greatest (and least) relation to lexical abilities, while the aggregated measure correlated to self-ratings that in turn correlated to performance in the weaker language. It is possible that including the separation in the HL-HEB-US group would have yielded more fine-grained results that may have mirrored effects found in the other groups. On the other hand, combining these measures in the latter groups may have coalesced into similar, less telling, effects as in the HL-HEB-US group.

In the HL-ENG-IL group, language use with friends was directly correlated with performance on both MINTs, such that the more a particular language was used with friends, the higher the score on the respective MINT. Meanwhile, no such direct relationship was observed in the HL-HEB-US group. Overall, the background questionnaire for the HL-HEB-US group assessed 14 different input measures, with the goal of teasing out potential effects and distinctions that might be overlooked by broader categories. However, we ultimately found that the majority of these factors did not correlate with lexical proficiency indices, while correlating strongly among themselves, leading us to question whether such granular views are necessary, as all factors are strongly interconnected. In fact, this was also the case for the other groups with fewer than 14 input indices. Thus, while it was interesting to consider such granularity as an exploration, the resulting interconnected associations suggest that such a detailed breakdown of language use is not needed, when a wider proxy measure—grouping together, for example, several input factors—can be applied. Taken together with the discussion about considering language use with the family as opposed to language use with different individual family members, this conclusion underscores the importance of finding an appropriate level of detail for a given set of variables, at the risk of overgeneralizing or grouping together distinct effects. Another aspect to consider when interpreting these findings is the precision and diverging scales used for the same factors across the different groups (see the example in section 3.2 of a question transforming from two scales of 5 into a scale of 3). Perhaps, had language use been measured comparatively across all groups using a wider scale, results would have swayed more toward or away from a particular factor.

Participants from the HL-HEB-US group additionally reported how important it was for them to maintain their Hebrew level, and the methods they use to maintain it, in addition to their perception of themselves as fully Israeli, fully American, a hyphenated hybrid, or otherwise. This latter identification was the weakest node in the network model, such that it had no association with participants’ lexical proficiency and was not clearly influenced by their language experience. This finding diverges from conclusions by Albirini (2014), who observed that a stronger sense of ethnic identity was tied to increased language use across contexts and to HL proficiency. This discrepancy highlights how the connection between ethnic identity and language proficiency might not be so clear-cut, as factors beyond lexical competence might be more central to a sense of identity depending on the particular culture or community. The knowledge of culturally-relevant terms might be more indicative of HL identity than the overall proficiency in the HL, and therefore lexical tests might include a subsection with culturally-relevant terms in addition to culturally-neutral ones (see Shabtaev et al., 2022).

In the first set of network models, considering only the common factors, the final model combining all five groups already deviated from the findings of each individual group. Juxtaposed with the larger model, these differences become all the more apparent. Based on this observation, and our findings from both the network models and the MINT results, we strongly advocate for considering language dynamics and contexts (i.e., heritage bilingualism vs. second language acquisition, the specific languages and settings, themselves, language status, etc.) when studying the bilingual language experience.

Of course, combining data from different questionnaires (each with its own foci and limitations, see Rothman et al., 2023) will always lead to “apples to oranges” comparisons to some degree, try as we may to drive them toward a common denominator. Over the last few years, increased efforts have been put toward more comprehensive questionnaires that would account for a wider range of bilingual experiences (see, for example, Tomić et al., 2023, HELEX questionnaire based on the LSBQ). Inevitably, or at least in the foreseeable future, studies will diverge in their focus and may want to adapt a given questionnaire for their particular aims, or for a unique context, returning us to our starting point of distinct, albeit similar, questionnaires. This is all the more pertinent for studies comparing language experience across contexts, as in the present work juxtaposing HL speakers and L2 learners in immersion and non-immersion contexts. Thus, using the data available, even when not identically matched, can help us at the very least highlight areas of interest for future, more targeted work. Network modeling can be a fitting step in this process.

### 5.3 Limitations and future directions

One limitation of our study was the absence of a L2-ENG-US group in the data, which would have served as a counterbalance to the L2-HEB-IL group and given us the fullest picture of this dyad. Intuitively, we would expect behavior to differ between these two groups, as the latter would be able to manage with relative ease in Israel by relying heavily on English, while the former, excluded group would not be able to rely analogously on Hebrew when navigating the United States. An additional limitation was the small sample size in each group, both on its own (i.e., the L2-HEB-IL group with 20 participants) and in conjunction with a relatively large set of collected variables (i.e., the HL-HEB-US group with 40 participants and 25 variables). This could leave the models vulnerable to biases within the data and less stable than they could otherwise have been. Thus, it is crucial to approach this study only as an exploration, suggesting intervariable relationships to consider in future work. In future analyses of this nature, we recommend including a stability analysis to further solidify extracted insights.

Another limitation was the crude scoring system used for several factors related to language use in different contexts, which was necessitated by the combination of multiple scales for assessing the same factor. Using a more granular scale may lead to a better representation of associations between the variables, which could impact the models.

The use of 4 questionnaires for the 5 groups was both a limitation and a feature. It acted as a limitation because the between-group comparisons were not completely matched and were strongly affected by data normalization, and we demonstrated how the set of factors taken into consideration could impact findings, especially as pertaining to the key central nodes in the network. However, the variety within the questionnaires also served as a feature, because we could show that even when considering different sets and numbers of variables, these variables consistently demonstrated interconnected relationships affecting each other as part of a system, and therefore should not be considered as fully independent measures.

## 6 Conclusion

In the present study, we set out to examine the bilingual lexical proficiency of five groups across the English-Hebrew dyad, and to explore interconnected relationships across networks of background and input factors. When looking at lexical proficiency, as measured by MINT scores in both languages, we found that all groups had a clearly dominant language, except the HL-ENG-IL group, which was balanced. We attribute this finding to a blend of the status of English in Israel and worldwide and academic reinforcement of the HL, and we suggest a closer examination of this phenomenon with HL-English in different contexts and also with academically supported HLs such as Spanish in the United States, in order to tease these explanatory factors apart. When considering conceptual vocabulary, all groups showed similar performance except the L2-HEB-US group. We attribute this effect to one (or some combination) of two possibilities: the participants in this group were on average older than in the others, suggesting that some items on the MINT may be less familiar to younger speakers, and the cultural neutrality of the Hebrew MINT assessment may have been overstated, as it appears to favor the North American context.

Our network models highlighted the differences in variable relationships between groups of bilinguals, pointing to the importance of considering these groups separately in their own right. In the networks, as in the MINTs, we saw a distinction between the groups as a function of bilingualism type and context. While some recent research has called for the consideration of a bilingual continuum when assessing language experience, nuances can be lost when we assess languages with highly different prestige levels. Therefore, it is important to account for these factors by distinguishing HL and L2 groups in accordance with their own context. Our methodology raised questions about which variables to include in such models, the effects of different scales, and the consequences of selecting certain levels of detail over others. Overall, we have shown how we can combine different measurement tools used separately and still extract meaningful exploratory insights, in the absence of perfectly matched questionnaires. We have demonstrated how network modeling enables researchers to more fully grasp the complexity of the bilingual experience by revealing complex interrelatedness between different background and input factors and showing us which connections may be worth further investigation. Therefore, we join previous calls for advancing this type of analysis in bilingualism research.

## Data availability statement

The datasets presented in this study can be found in online repositories. The names of the repository/repositories and accession number(s) can be found at: https://osf.io/p5ckj/?view_only=186fbcbb6f4e476bbea64e7b5f443627.

## Ethics statement

The studies involving humans were approved by the Institutional Review Board of Bar Ilan University. The studies were conducted in accordance with the local legislation and institutional requirements. The participants provided their written informed consent to participate in this study.

## Author contributions

CF: Conceptualization, Data curation, Formal analysis, Investigation, Methodology, Project administration, Software, Validation, Visualization, Writing – original draft, Writing – review & editing. AL: Data curation, Investigation, Writing – review & editing. SB: Data curation, Investigation, Writing – review & editing. NM: Conceptualization, Funding acquisition, Supervision, Writing – review & editing.
