# Supplementary material for: Modeling lexical abilities of heritage language and L2 speakers of Hebrew and English in Israel and the United States: a network approach
Source: Front Psychol. 2024 Apr 24;15:1331801. doi: 10.3389/fpsyg.2024.1331801 (PMC11110673; doi:10.3389/fpsyg.2024.1331801)
Supplement: Supplementary file 1 [file Table_1.DOCX]

Supplementary Material

# Variables Collected (and used in the network model) from each Questionnaire/Group

|  | Questionnaire | | | |
| --- | --- | --- | --- | --- |
| Variable | A (L2-HEB-US) | B (L2-HEB-IL) | C (HL-ENG-IL, L2-ENG-IL) | D (HL-HEB-US) |
| Age | x | x | x | x |
| Age of Onset of Bilingualism (AoB) | x | x | x | x |
| Age at Immigration |  | x | x |  |
| Language Use with Mother |  |  | x |  |
| Language Use with Father |  |  | x |  |
| Language Use with Siblings |  |  | x |  |
| Language Use with Friends |  |  | x | x |
| Mother’s Years of Education | x |  |  |  |
| Father’s Years of Education | x |  |  |  |
| Participant’s Years of Education |  | x |  |  |
| Hebrew Foreign Accent Rating |  | x |  |  |
| Self-Rated Hebrew Level |  | x |  | x |
| Years Living in Israel |  | x |  |  |
| Daily English Percentage |  | x |  |  |
| Self-Rated English Level |  |  |  | x |
| Hebrew Narrative (# unique Hebrew tokens produced) |  |  |  | x |
| Number of maintenance methods |  |  |  | x |
| Visit Frequency to Israel |  |  |  | x |
| Language Use with Immediate Family |  |  |  | x |
| Language Use with Extended Family |  |  |  | x |
| Language Use at Work |  |  |  | x |
| Language Use Day-to-Day |  |  |  | x |
| Language Use for Religion |  |  |  | x |
| Language Use for Media |  |  |  | x |
| Language Use ages 0-5 |  |  |  | x |
| Language Use ages 6-12 |  |  |  | x |
| Language Use ages 13-17 |  |  |  | x |
| Language Use ages 18+ |  |  |  | x |
| Importance of Maintaining HL |  |  |  | x |
| Identity |  |  |  | x |

# Scales and Formulations for each Variable in each Questionnaire/Group

|  | Questionnaire | | | |
| --- | --- | --- | --- | --- |
| Variable | A (L2-HEB-US) | B (L2-HEB-IL) | C (HL-ENG-IL, L2-ENG-IL) | D (HL-HEB-US) |
| Age | Years | Years | Years | Years |
| Age of Onset of Bilingualism (AoB) | Years | Years | Years | Years |
| Age at Immigration |  | Years | Years |  |
| Language Use with Mother |  |  | (A) Only Hebrew-Mostly Hebrew- Half Hebrew / Half English-Mostly English- Only English  (B) English-Hebrew-Both  Converted into: 1-English (Mostly/Only) 2-Both English and Hebrew (Half/Half) 3-Hebrew (Mostly/Only Hebrew) |  |
| Language Use with Father |  |  | (A) Only Hebrew-Mostly Hebrew- Half Hebrew / Half English-Mostly English- Only English  (B) English-Hebrew-Both  Converted into: 1-English (Mostly/Only) 2-Both English and Hebrew (Half/Half) 3-Hebrew (Mostly/Only Hebrew) |  |
| Language Use with Siblings |  |  | (A) Only Hebrew-Mostly Hebrew- Half Hebrew / Half English-Mostly English- Only English  (B) English-Hebrew-Both  Converted into: 1-English (Mostly/Only) 2-Both English and Hebrew (Half/Half) 3-Hebrew (Mostly/Only Hebrew) |  |
| Language Use with Friends |  |  | (A) Only Hebrew-Mostly Hebrew- Half Hebrew / Half English-Mostly English- Only English  (B) English-Hebrew-Both  Converted into: 1-English (Mostly/Only) 2-Both English and Hebrew (Half/Half) 3-Hebrew (Mostly/Only Hebrew) | Frequency of Hebrew Use: Never-Rarely-Sometimes-Often-Always  Converted into: 1-English (Never/Rarely) 2-Both English and Hebrew (Sometimes) 3-Hebrew (Often/Always) |
| Mother’s Years of Education | High School-Post-High-School-Certificate- Bachelor’s Degree- Master’s Degree-Doctorate-Post-Doctorate  Converted to: Years |  |  |  |
| Father’s Years of Education | High School-Post-High-School-Certificate- Bachelor’s Degree- Master’s Degree-Doctorate-Post-Doctorate  Converted to: Years |  |  |  |
| Participant’s Years of Education |  | Years |  |  |
| Hebrew Foreign Accent Rating |  | X |  |  |
| Self-Rated Hebrew Level |  | 1-7 rating on reading, writing, comprehending and speaking  (converted to 0-100 after getting the summed total out of 28) |  | 1-5 (converted to 0-100) |
| Years Living in Israel |  | Years |  |  |
| Daily English Percentage |  | x |  |  |
| Self-Rated English Level |  |  |  | 1-5 (converted to 0-100) |
| Hebrew Narrative |  |  |  | Number unique Hebrew tokens produced |
| Number of maintenance methods |  |  |  | Number of different methods marked (from the checklist) |
| Visit Frequency to Israel |  |  |  | I have not visited in the last decade- less than once every few years- once every few years – once a year- more than once a year  Converted into a 1-5 scale |
| Language Use with Immediate Family |  |  |  | Frequency of Hebrew Use: Never-Rarely-Sometimes-Often-Always  Converted into: 1-English (Never/Rarely) 2-Both English and Hebrew (Sometimes) 3-Hebrew (Often/Always) |
| Language Use with Extended Family |  |  |  | Frequency of Hebrew Use: Never-Rarely-Sometimes-Often-Always  Converted into: 1-English (Never/Rarely) 2-Both English and Hebrew (Sometimes) 3-Hebrew (Often/Always) |
| Language Use at Work |  |  |  | Frequency of Hebrew Use: Never-Rarely-Sometimes-Often-Always  Converted into: 1-English (Never/Rarely) 2-Both English and Hebrew (Sometimes) 3-Hebrew (Often/Always) |
| Language Use Day-to-Day |  |  |  | Frequency of Hebrew Use: Never-Rarely-Sometimes-Often-Always  Converted into: 1-English (Never/Rarely) 2-Both English and Hebrew (Sometimes) 3-Hebrew (Often/Always) |
| Language Use for Religion |  |  |  | Frequency of Hebrew Use: Never-Rarely-Sometimes-Often-Always  Converted into: 1-English (Never/Rarely) 2-Both English and Hebrew (Sometimes) 3-Hebrew (Often/Always) |
| Language Use for Media |  |  |  | Frequency of Hebrew Use: Never-Rarely-Sometimes-Often-Always  Converted into: 1-English (Never/Rarely) 2-Both English and Hebrew (Sometimes) 3-Hebrew (Often/Always) |
| Language Use ages 0-5 |  |  |  | Hebrew-English-Both-Other  Converted into: 1-English 2-Both English and Hebrew 3-Hebrew |
| Language Use ages 6-12 |  |  |  | Hebrew-English-Both-Other  Converted into: 1-English 2-Both English and Hebrew 3-Hebrew |
| Language Use ages 13-17 |  |  |  | Hebrew-English-Both-Other  Converted into: 1-English 2-Both English and Hebrew 3-Hebrew |
| Language Use ages 18+ |  |  |  | Hebrew-English-Both-Other  Converted into: 1-English 2-Both English and Hebrew 3-Hebrew |
| Importance of Maintaining HL |  |  |  | 1 (not important at all) -5 (very important) |
| Identity |  |  |  | Israeli, American, Israel-American, Jewish, Other  Converted into: 1: American 2: Jewish 3: Jewish Israeli American 4: Israeli American 5:Israeli |

# Conceptual Vocabulary Scores by Item, Group

| Item Number | HLE-SLH-IL | HLH-SLE-US | L1E-L2H-IL | L1E-L2H-US | L1H-L2E-IL |
| --- | --- | --- | --- | --- | --- |
| 1 | 100% | 100% | 100% | 100% | 100% |
| 2 | 100% | 100% | 100% | 100% | 100% |
| 3 | 100% | 100% | 100% | 100% | 100% |
| 4 | 100% | 100% | 100% | 100% | 100% |
| 5 | 100% | 100% | 100% | 100% | 100% |
| 6 | 100% | 100% | 100% | 100% | 100% |
| 7 | 100% | 100% | 100% | 100% | 100% |
| 8 | 100% | 100% | 100% | 100% | 100% |
| 9 | 100% | 100% | 100% | 100% | 100% |
| 10 | 100% | 100% | 100% | 100% | 100% |
| 11 | 100% | 100% | 100% | 100% | 100% |
| 12 | 100% | 100% | 100% | 100% | 100% |
| 13 | 100% | 100% | 100% | 100% | 100% |
| 14 | 100% | 100% | 100% | 100% | 100% |
| 15 | 100% | 100% | 100% | 100% | 100% |
| 16 | 100% | 100% | 100% | 100% | 100% |
| 17 | 100% | 100% | 100% | 100% | 100% |
| 18 | 100% | 100% | 100% | 100% | 100% |
| 19 | 100% | 100% | 100% | 100% | 100% |
| 20 | 94% | 100% | 100% | 100% | 76% |
| 21 | 100% | 100% | 100% | 100% | 100% |
| 22 | 100% | 100% | 100% | 100% | 100% |
| 23 | 100% | 100% | 100% | 100% | 100% |
| 24 | 100% | 100% | 100% | 100% | 100% |
| 25 | 100% | 100% | 100% | 100% | 100% |
| 26 | 100% | 100% | 100% | 100% | 100% |
| 27 | 100% | 100% | 100% | 100% | 100% |
| 28 | 100% | 100% | 100% | 100% | 100% |
| 29 | 100% | 100% | 100% | 100% | 98% |
| 30 | 100% | 100% | 100% | 100% | 100% |
| 31 | 100% | 100% | 100% | 100% | 98% |
| 32 | 100% | 100% | 100% | 100% | 100% |
| 33 | 100% | 100% | 100% | 100% | 100% |
| 34 | 96% | 88% | 100% | 100% | 98% |
| 35 | 100% | 100% | 100% | 100% | 100% |
| 36 | 100% | 100% | 100% | 100% | 100% |
| 37 | 98% | 100% | 100% | 100% | 100% |
| 38 | 98% | 100% | 100% | 100% | 100% |
| 39 | 100% | 100% | 100% | 96% | 98% |
| 40 | 100% | 100% | 100% | 100% | 100% |
| 41 | 100% | 100% | 100% | 100% | 100% |
| 42 | 100% | 100% | 100% | 96% | 100% |
| 43 | 98% | 100% | 100% | 100% | 100% |
| 44 | 100% | 100% | 100% | 100% | 100% |
| 45 | 98% | 100% | 100% | 96% | 98% |
| 46 | 100% | 93% | 100% | 96% | 98% |
| 47 | 100% | 100% | 100% | 100% | 100% |
| 48 | 100% | 100% | 100% | 100% | 100% |
| 49 | 77% | 75% | 95% | 96% | 82% |
| 50 | 96% | 100% | 100% | 100% | 94% |
| 51 | 100% | 98% | 100% | 100% | 100% |
| 52 | 88% | 73% | 100% | 89% | 82% |
| 53 | 100% | 100% | 100% | 100% | 100% |
| 54 | 92% | 85% | 100% | 93% | 80% |
| 55 | 100% | 100% | 100% | 100% | 100% |
| 56 | 100% | 95% | 100% | 100% | 100% |
| 57 | 100% | 100% | 100% | 100% | 98% |
| 58 | 92% | 70% | 90% | 93% | 96% |
| 59 | 100% | 90% | 100% | 100% | 100% |
| 60 | 98% | 85% | 100% | 100% | 98% |
| 61 | 40% | 65% | 65% | 86% | 40% |
| 62 | 73% | 93% | 85% | 100% | 86% |
| 63 | 35% | 45% | 70% | 50% | 44% |
| 64 | 23% | 13% | 60% | 39% | 16% |
| 65 | 27% | 58% | 30% | 54% | 40% |
| 66 | 21% | 58% | 85% | 64% | 32% |
| 67 | 27% | 53% | 85% | 61% | 44% |
| 68 | 8% | 15% | 30% | 64% | 8% |

# Centrality Analyses


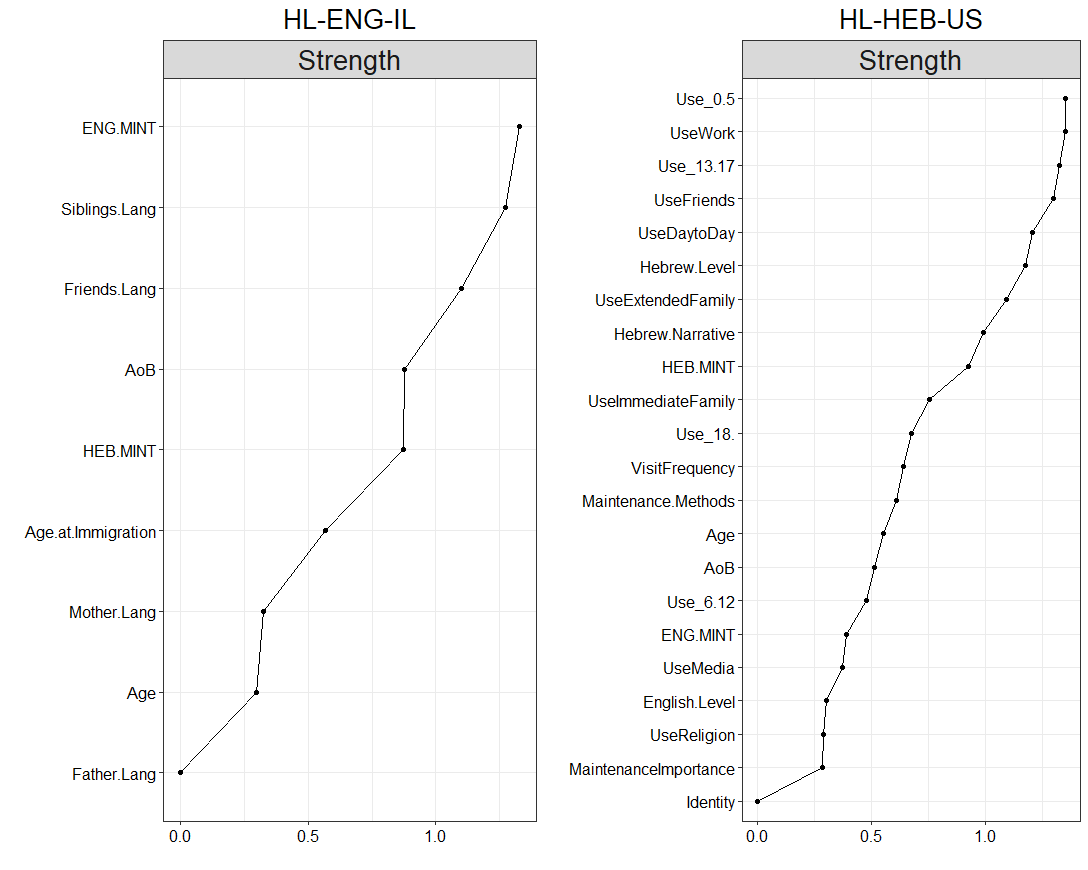


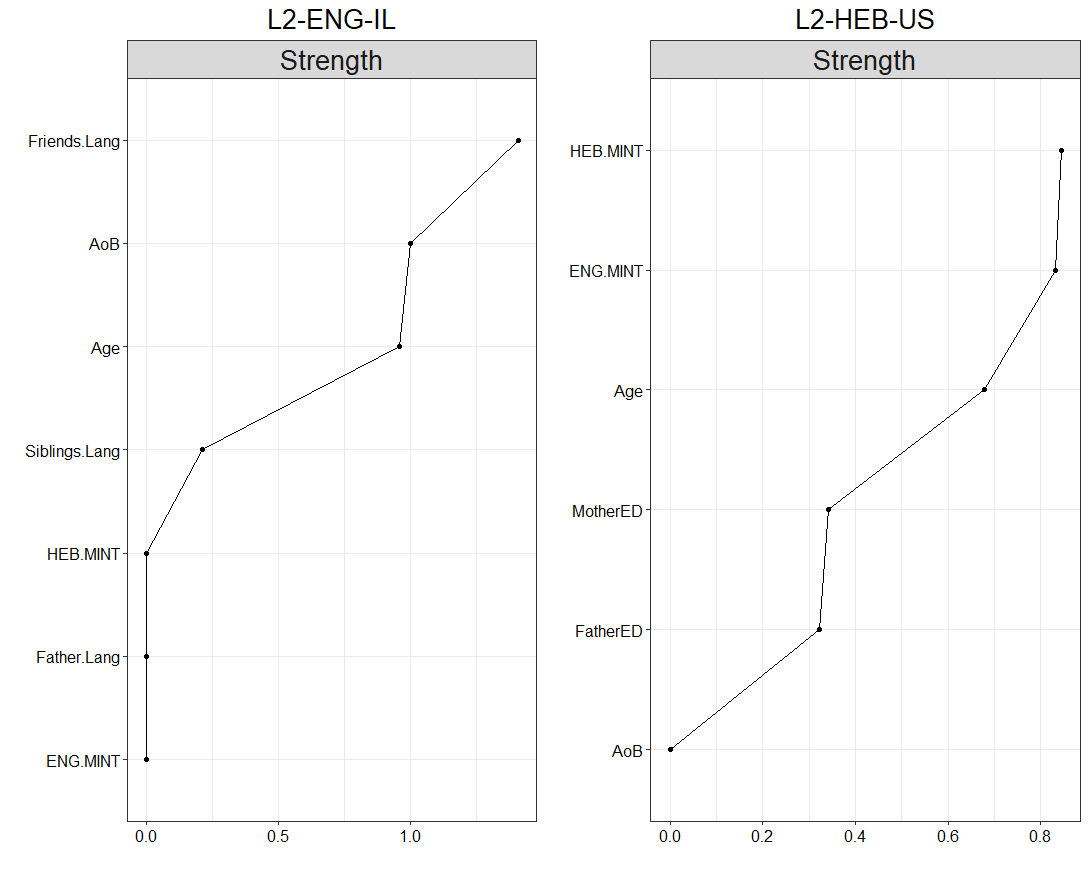


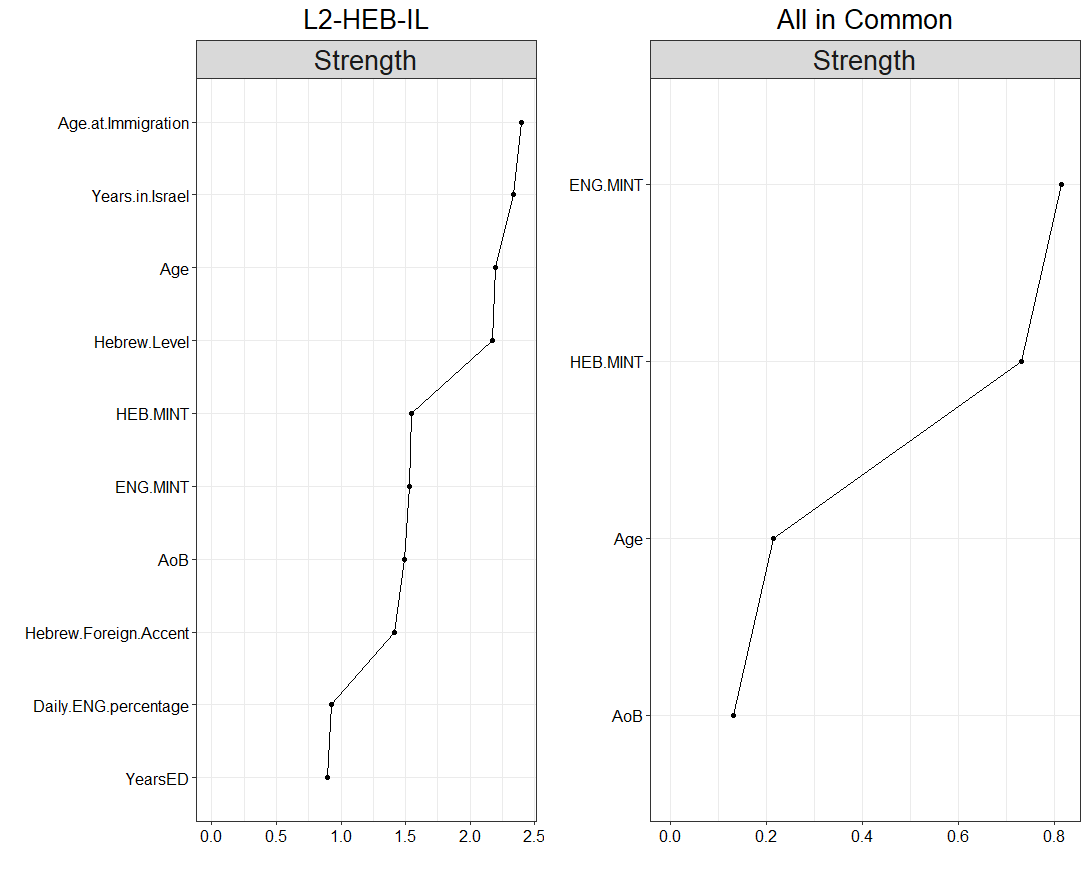


# Questionnaire A

Participant Number:
First Name:
First Letter of Last Name:
Age:
Gender: Male / Female
Country of Birth:
What language do you consider to be your mother tongue?
Growing up, what language did your mother use when speaking to you? Only Hebrew / Mostly Hebrew / Both Hebrew and English / Mostly English / Only English / Other
Growing up, what language did your father use when speaking to you? Only Hebrew / Mostly Hebrew / Both Hebrew and English / Mostly English / Only English / Other
Do you know any other languages? If so, what are they?
If you answered yes to the question above, how did you learn that other language? For example at school, in the neighborhood, from TV, etc.
Have you learned Hebrew? Yes/No/A little
If you answered YES or A LITTLE to the question above, at what age did you begin to learn Hebrew?
Your current occupation: High school student / post high-school / university student / other
What is the highest level of schooling that your mother has completed? High school / post high school certificate / Bachelor’s degree / Master’s Degree / Doctorate / Post-doctorate
What is the highest level of schooling that your father has completed? High school / post high school certificate / Bachelor’s degree / Master’s Degree / Doctorate / Post-doctorate

# Questionnaire B

Participant's code:
Age:
Gender: Male / Female / Other
How many years of formal education do you have (school, university etc.)?
Please check your highest educational achievement (or the approximate Israeli equivalent to a degree obtained in another country): Less than high school / high school / non academic professional training / BA / MA / PhD / Other
Profession:
Country of Origin:
Country of Residence:
If your country of origin is other than Israel, at what age did you immigrate to Israel?
Total number of years living in Israel:
At what age did you acquire English?
At what age did you acquire Hebrew?
What language do you usually speak with your mother: Hebrew / English / Both / Other
What language do you usually speak with your father: Hebrew / English / Both / Other
What language do you usually speak with your siblings: Hebrew / English / Both / Other
What language do you usually speak with your partner: Hebrew / English / Both / Other
What language do you usually speak with your children: Hebrew / English / Both / Other
What language do you usually speak with your friends: Hebrew / English / Both / Other
On a scale of 1-7,  select your level of proficiency in reading, writing, comprehending, and  speaking English.
On a scale of 1-7,  select your level of proficiency in reading, writing, comprehending, and  speaking Hebrew.
In your perception, do you have a foreign accent in English? 1-7
In your perception, do you have a foreign accent in Hebrew? 1-7
Estimate, in terms of percentages, how often you use English, Hebrew, and other languages per day (in all daily activities combined): Intervals of 10%

# Questionnaire C

The common questions from Questionnaires A and B

# Questionnaire D

First three letters of your name
Age
Gender: Male / Female
Country of Birth:
Age of immigration to the US
How many of your parents speak Hebrew: One / Both
Level of English (overall): 1-5
Level of Hebrew (overall): 1-5
Level of Hebrew (reading): 1-5
Level of Hebrew (writing): 1-5
How often do you visit Israel? I have never visited / I have not visited in the last decade / Less than once few years / once every few years / Once a year / More than once a year
How important is it for you to maintain your Hebrew? 1-5
Which of the following methods have you used to maintain your Hebrew? (Check all that apply) Attended Jewish day school / Attended Hebrew Language Sunday School / Taken Hebrew classes in high school or college / Visited family in Israel (for more than 1 month at a time) / Participated in programs in Israel (for more than 1 month at a time) / Participated in Hebrew programming offered at your university (Hillel, Mishelanu, etc) / Participated in Hebrew-language events in your local community / Attended Hebrew-language religious services / Other
What are your motivations (if any) for maintaining Hebrew?
How often do you use Hebrew with your immediate family? 1-5
How often do you use Hebrew with your extended family? 1-5
How often do you use Hebrew with your friends? 1-5
How often do you use Hebrew at work? 1-5
How often do you use Hebrew day-to-day? 1-5
How often do you use Hebrew at religious services? 1-5
How often do you use Hebrew for media consumption? 1-5
Which languages did you use at age 0-5: Hebrew / English / Both / Other
Which languages did you use at age 6-12: Hebrew / English / Both / Other
Which languages did you use at age 13-17: Hebrew / English / Both / Other
Which languages did you use at age 18+: Hebrew / English / Both / Other
Which identity do you feel best describes you? Israeli / American / Israel-American / Jewish
